# Efficient Preparation of Chitooligosaccharide With a Potential Chitosanase Csn-SH and Its Application for Fungi Disease Protection

**DOI:** 10.3389/fmicb.2021.682829

**Published:** 2021-06-17

**Authors:** Dandan Cui, Jin Yang, Bosi Lu, Hong Shen

**Affiliations:** ^1^College of Natural Resources and Environment, South China Agricultural University, Guangzhou, China; ^2^Guangdong Provincial Key Laboratory of Eco-Circular Agriculture, Guangzhou, China

**Keywords:** chitosanase, *Bacillus atrophaeus*, chitooligosaccharide, antifungal activity, hydrolysis rate

## Abstract

Chitosanase plays a vital role in bioactive chitooligosaccharide preparation. Here, we characterized and prepared a potential GH46 family chitosanase from *Bacillus atrophaeus* BSS. The purified recombinant enzyme Csn-SH showed a molecular weight of 27.0 kDa. Csn-SH displayed maximal activity toward chitosan at pH 5.0 and 45°C. Thin-layer chromatography and electrospray ionization–mass spectrometry indicated that Csn-SH mainly hydrolyzed chitosan into (GlcN)_2_, (GlcN)_3_, and (GlcN)_4_ with an endo-type cleavage pattern. Molecular docking analysis demonstrated that Csn-SH cleaved the glycoside bonds between subsites −2 and + 1 of (GlcN)_6_. Importantly, the chitosan hydrolysis rate of Csn-SH reached 80.57% within 40 min, which could reduce time and water consumption. The hydrolysates prepared with Csn-SH exhibited a good antifungal activity against *Magnaporthe oryzae* and *Colletotrichum higginsianum*. The above results suggested that Csn-SH could be used to produce active chitooligosaccharides efficiently that are biocontrol agents applicable for safe and sustainable agricultural production.

## Introduction

Chitin is the second largest natural polysaccharide after cellulose and exists widely in arthropods exoskeletons, fungi, and insect intestinal mucosa. Because of its insolubility in common solvents, chitin is still a rarely utilized natural biomass (Luo et al., 2020). Chitosan is a partially or completely deacetylated derivative of chitin that consists mainly of D-glucosamine (GlcN) linked with β-1,4-glycosidic bonds and contains a small amount of N-acetyl-D-glucosamine (GlcNAc) (Zheng et al., 2021). Chitosan exhibits good biodegradability and biocompatibility and is nontoxic and soluble in weak acid. Thus, it has been widely used in gene therapy, drug delivery, wound repair, and microbial growth inhibition (Yang et al., 2020). Nevertheless, chitosan is insoluble in water and soluble only in some inorganic and organic acids, such as acetic, hydrochloric, lactic, and citric acids, among which acetic acid is the most commonly used (Nascimento et al., 2020). Chitooligosaccharides (COSs), are degradation products of chitosan consisting of depolymerized derivatives of chitosan with a degree of polymerization (DP) ranging from 2 to 10 (Benchamas et al., 2021). COSs are nontoxic, water-soluble, and biocompatible and have been widely used as antitumor (Jiang et al., 2020), drug-delivery (Wang et al., 2021a), antimicrobial (Silva et al., 2021), antifungal (Zhou et al., 2020), immune-activating (Zhang et al., 2021), and food preservation agents (Dutta et al., 2012).

Chitosan has been shown to be degraded to COSs by chemical methods or enzymatic processes. However, chemical methods have many drawbacks, such as polluting the environment, harming the physicochemical properties of COSs, and presenting high costs. In contrast, enzymatic hydrolysis presents many advantages, such as an absence of damage to the active group structure, safety, less environmental pollution, high yields, and controllability. Many enzymes have been reported to hydrolyze chitosan, such as cellulase, papain, pepsin, pectinase, and lipase, and chitosanase (EC. 3.2.1.132) hydrolyzes chitosan specifically (Wang et al., 2021b).

According to amino acid sequence similarity, chitosanases are divided into seven families in the CAZy database, including GH3, GH5, GH7, GH8, GH46, GH75, and GH80, among which the GH46, GH75, and GH80 families only contain chitosanases (Viens et al., 2015). GH46 is currently the most extensively studied family of chitosanase and mainly consists of enzymes derived from *Bacillus subtilis*, *Pseudomonas* sp., *Streptomyces* sp., *Kitasatospora* sp., and other bacterial genera (Viens et al., 2015). Owing to the low enzymatic activities of most original strains, cloned and exogenously expressed chitosanases have attracted extensive attention of people. In recent years, the exogenous expression of chitosanase derived from *Bacillus*, *Staphylococcus*, *Microbacterium*, *Streptomyces*, and other bacterial genera has been reported, among which *Streptomyces* and *Bacillus* have provided the greatest numbers of these enzymes. However, there are few reports regarding the heterologous expression of chitinase and chitosanase from *Bacillus atrophaeus*.

The *B. atrophaeus* BSS genome sequences were submitted to the database of National Center for Biotechnology Information (NCBI) in 2014. A chitosanase gene was annotated in the genome. In the present study, the chitosanase gene *csn* from *B. atrophaeus* BSS was cloned and heterologously expressed in *Escherichia coli* BL21 (DE3). The physicochemical properties, hydrolytic characteristics, and hydrolysis rate of the recombinant chitosanase Csn-SH were investigated. Furthermore, the antifungal activities of the COSs prepared with Csn-SH were also evaluated.

## Materials and Methods

### Materials

Colloidal chitosan (degree of deacetylation [DD] ≥ 85%) and water-soluble chitosan (DD ≥ 95%) were purchased from Qingdao BZ Oligo Biotech Co., Ltd. (Qingdao, China). (GlcN)_2__–__6_ (DP2–6) were purchased from Huizhou Long Dragon Biotechnology Co., Ltd. (Huizhou, China). Colloidal chitin and carboxymethyl cellulose (CMC) were purchased from Aladdin (Shanghai, China). COSs (DP2–7) were purchased from Guangzhou SGY Agricultural Science and Technology Co., Ltd. (Guangzhou, China). *Magnaporthe oryzae* and *Colletotrichum higginsianum* were kindly provided by Prof. Yunfeng Li (College of Plant Protection) and Prof. Shujie Feng (College of Horticulture), South China Agricultural University, Guangzhou, China, respectively.

### Gene Cloning and Sequence Analysis

A new isolated *B. atrophaeus* strain BSS was identified, and the genome sequences were submitted to NCBI database by H. E. Daligault et al. in 2014. Based on the genome information of *B. atrophaeus* BSS (GenBank: CP007640.1), a GH46 chitosanase gene *csn* was annotated. The open reading frame was mapped by ORF finder.^1^ Prediction of signal peptides was performed using the SignalP 5.0.^2^ The analysis of conserved domain was carried out by the Conserved Domain Database of NCBI. The theoretical molecular weight (Mw) and isoelectronic point (pI) were computed using the tool of ExPASy ProtParam without the signal peptide.^3^ Multiple sequence alignment was performed using Clustal Omega^4^ and obtained using DNAMAN (9.0) and ESPript 3.0^5^ after the removal of signal peptide. Phylogenetic and molecular evolutionary analyses were conducted by using MEGA 7.0 software.

To explore the potential catalytic function of *csn*, we optimized the gene codon according to the *E. coli* codon preference. The optimized gene was chemically synthesized by Sangon (Shanghai, China) without signal peptide and inserted into plasmid pET-28a (+). The recombinant plasmid was transformed to *E. coli* BL21 (DE3) competent cells by heat shock method for expression of the chitosanase. The positive transformants were selected on Luria–Bertani (LB) agar plates supplemented with 50 μg/mL kanamycin.

### Gene Expression and Purification

The recombinant *E. coli* BL21 (DE3) cells were cultivated in LB medium containing 50 μg/mL kanamycin at 37°C in a shaker (180 revolutions/min). When OD_600_ of the culture reached 0.6, isopropyl-β-D-thiogalactopyranoside (IPTG) was added into the culture at a final concentration of 0.01–1 mM. The induced cultures were further grown at 20, 25, 30, or 35°C for 12 h, and the cells were harvested by centrifugation at 10,000*g* for 10 min, washed twice with 20 mM sodium phosphate buffer (pH 7.0), and stored at −20°C.

The harvested cells were resuspended in sodium phosphate buffer (20 mM, pH 7.0) and disrupted by sonication, and then the debris was removed by centrifugation at 10,000*g* for 10 min at 4°C. The resulting crude extract was purified by Ni-NTA column (Sangon, China) according to the protocol, and Csn-SH was collected and carried out by sodium dodecyl sulfate–polyacrylamide gel electrophoresis (SDS-PAGE). The protein concentration was determined using the Bradford method, and bovine serum albumin was used as the standard (Luo et al., 2020).

### Enzyme Activity Assay

Chitosanase assays were performed according to the method (Sun et al., 2020). Briefly, 100 μL properly diluted enzyme solution was mixed with 900 μL of 0.5 % (wt/vol) colloidal chitosan, and then 1 mL sodium acetate buffer (20 mM, pH 5.0) was added to the above mixture. The reaction mixture was incubated at 40°C for 10 min. The reaction was quenched by adding 1 mL dinitrosalicylic acid solution and then incubated in boiling water for 10 min. The reducing sugar in the supernatant was determined by measuring absorbance at 520 nm with D-glucosamine as the standard. All the experiments were performed in triplicate. One unit (U) of chitosanase activity was defined as the amount of enzyme needed to liberate 1 μmol D-glucosamine-equivalent–reducing sugar per minute in the above assay conditions.

### Characterization of the Purified Csn-SH

Colloidal chitosan (0.5%, wt/vol) was used as the substrate to determine the enzymatic characterization of Csn-SH. The optimal pH for Csn-SH activity was determined by measuring the enzyme activity from pH 2.5 to 6.0 using 20 mM various buffers as follows: Gly-HCl buffer, pH 2.5 to 3.5; sodium acetate buffer, pH 3.5 to 6.0. The reaction mixture consisted of 100 μL Csn-SH, 900 μL chitosan, and 1 mL buffer. To determine pH stability, 100 μL Csn-SH was added to 900 μL different 20 mM buffers without substrate at 45°C for 1 h. The buffers used were as follows: Gly-HCl buffer, pH 2.5–3.5; sodium acetate buffer, pH 3.5–6.0; sodium phosphate buffer, pH 6.0–8.0; and Tris–HCl buffer, pH 8.0–10.0. After preincubation, the residual chitosanase activity was measured under standard assay conditions.

The optimal temperature for Csn-SH activity was examined by incubating the enzyme with chitosan at 20°C to 80°C in 20 mM sodium acetate buffer (pH 5.0). The enzymatic thermostability was evaluated by measuring the residual activity of Csn-SH incubated at temperature ranging from 30°C to 60°C in 20 mM sodium acetate buffer (pH 5.0) for 0.5 to 2 h. The residual enzymatic activity was tested in standard assay conditions.

The effects of metal cations (NaCl, KCl, Li_2_SO_4_, MgCl_2_, CaCl_2_, CuCl_2_, MnCl_2_, CoCl_2_, ZnSO_4_, FeSO_4_, PbSO_4_, FeCl_3_, and AlCl_3_) and chemical (EDTA) on the enzymatic activity of Csn-SH were determined at the final concentration of 1 and 5 mM in the reaction mixture at 45°C for 10 min. The catalytic activity without any chemicals was used as the control, and the activity was defined as 100%.

### Substrate Specificity

Substrate specificity of Csn-SH was determined in 20 mM sodium acetate buffer (pH 5.0) at 45°C for 10 min. The tested substrates (0.5% wt/vol) included colloidal chitosan (DD ≥ 85%), water-soluble chitosan (DD ≥ 95%), colloidal chitin, and CMC.

### Determination of the Kinetic Parameters of Csn-SH

The kinetic parameters of Csn-SH were analyzed under optimal conditions as described previously and used various concentrations of colloidal chitosan. [S] indicated the chitosan concentrations, which ranged from 0.2 to 2.0 mg/mL with an interval of 0.2. The reactions were performed at 45°C for 10 min in sodium acetate buffer (20 mM, pH 5.0). The *K*_*m*_ and *V*_*max*_ values were calculated by the Lineweaver–Burk equation (Zhou et al., 2020).

### Hydrolysis Pattern of Csn-SH

The hydrolysis properties of Csn-SH were investigated by using colloidal chitosan and COSs viz. (GlcN)_2_, (GlcN)_3_, (GlcN)_4_, (GlcN)_5_, and (GlcN)_6_ as substrates. The hydrolysates prepared with Csn-SH were analyzed through thin-layer chromatography (TLC) and electrospray ionization–mass spectrometry (ESI-MS). In brief, 100 μL Csn-SH (20 U/mL) was mixed with 900 μL 1% (wt/vol) different substrates including (GlcN)_2_, (GlcN)_3_, (GlcN)_4_, (GlcN)_5_, (GlcN)_6_, and colloidal chitosan, respectively, and 1 mL sodium acetate buffer (20 mM, pH 5.0) was added into the mixture and incubated at 45°C. Samples were withdrawn at different times and immediately boiled for 10 min before centrifuged at 12,000*g* for 5 min. The supernatant samples were spotted in a TLC plate (Silica gel 60 F254 aluminum sheet; Merck, Darmstadt, Germany), developed in isopropanol/ammonium (2:1, vol/vol) solvent, and sprayed with 0.1% ninhydrin reagent (dissolved in ethanol). The hydrolysates were visualized by heating the plate at 110°C in an oven for 5 min. ESI-MS studies were performed in positive-ion mode with a ratio of mass to charge in the range of 50 to 2,000 (*m/z*). The scan mode was used under a capillary needle at 3.5 kV, and the ion source temperature was kept at 180°C.

### Homology Modeling and Molecular Docking

Homology models of Csn-SH were generated using the Modeller 9.19 with the chitosanase from *B. subtilis* MY002 (PDB: 7C6C; identity: 91.3%) as the template (Li et al., 2021). Molecular docking between Csn-SH and (GlcN)_6_ was performed by Autodock (Lyu et al., 2014). Then, the Csn-SH complexed with (GlcN)_6_ was superimposed on the structure of chitosanase CsnMY002 from *B. subtilis* MY002 (PDB: 7C6C) using PyMOL.

### Inhibitory Effects of COSs on Fungi

The inhibitory effects of COSs prepared with Csn-SH on the phytopathogenic fungi *M. oryzae* and *C. higginsianum* were investigated. The fungi preserved on an inclined surface were inoculated onto the potato dextrose agar (PDA) plate and cultured at 28°C for 3 days to obtain a fresh colony. Then, a sterile blade was used to cut the edge of the colony to obtain the fungus cakes with a diameter of 6 mm. The mycelial side of the cake was inoculated down to the center of PDA plates separately containing 0.1, 0.5, 1.0, 2.0, 4.0, and 8.0 mg/mL COSs. COSs-Csn indicated the COSs prepared with Csn-SH, whereas COSs-SGY represented COSs purchased from Guangzhou SGY Agricultural Science and Technology Co., Ltd. For fungi *M. oryzae*, the plates were cultured at 28°C for 6 days, and the plates of *C. higginsianum* were cultured at 25°C for 7 days. The PDA plate without COSs was used as the control. Finally, the diameters of the fungal mycelia were measured. The growth inhibition rate was calculated according to the previous study (Zhou et al., 2020).

## Results and Discussion

### Sequence Analysis of Csn-SH

The *csn* gene from *B. atrophaeus* strain BSS genome (GenBank: CP007640.1) contained an 825-bp open reading frame, which encoded a chitosanase (Csn-SH) of 274 amino acids. A 32-amino-acid signal peptide was predicted at the N-terminus via SignalP analysis. Domain structure prediction analysis indicated that Csn-SH was a single-domain protein with a GH family 46 catalytic regions between residues 38 and 261. The theoretical Mw and pI of Csn-SH were 27.4 kDa and 5.62 without signal peptide, respectively.

**FIGURE 1:**
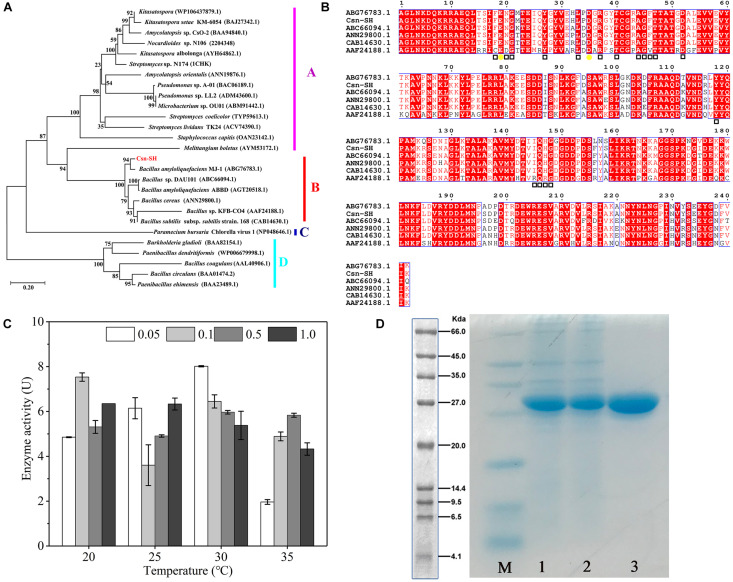
Sequence analysis, expression, and purification of Csn-SH. **(A)** The neighbor-joining tree shows phylogenetic relationships between Csn-SH and other GH46 family members. The scale bar indicates the average number of amino acid substitutions per site. The bootstrap test of the tree was performed with 1,000 replications. **(B)** Sequence alignment of Csn-SH with other GH46 family members. The catalytic sites and sugar-binding sites were marked with yellow circles and hollow squares, respectively. The sequence of Csn-SH did not include the 32-amino-acid residues (signal peptide) at N-terminus. **(C)** The enzyme activities of Csn-SH induced by different concentrations of IPTG at different temperatures, whereas 0.05, 0.1, 0.5, and 1.0 indicated the concentration (mM) of IPTG. **(D)** SDS-PAGE analysis of purified Csn-SH. M, molecular marker; 1 and 2, crude enzymes induced by 0.05 mM IPTG at 30°C; 3, purified Csn-SH.

According to the sequence alignments and phylogenetic tree analysis, the chitosanases of GH46 family were grouped into five clusters A, B, C, D, and E, and almost all chitosanases in cluster B were derived from Bacilli (Viens et al., 2015). The phylogenetic tree analysis results indicated that Csn-SH was a novel member of cluster B (Figure 1A) and showed higher homology with the chitosanase from *Bacillus amyloliquefaciens* MJ-1 (Hong and Kang, 2006). Glutamic acid (E) and aspartic acid (D) are two conserved catalytic residues in the GH46 family (Takasuka et al., 2014); the corresponding residues of Csn-SH were E19 and D35 (Figure 1B, labeled with yellow circles). Figure 1B also showed that multiple substrate-binding sites (labeled with hollow squares) played important roles in the substrate preference of Csn-SH (Yang et al., 2019).

### Expression and Purification of Csn-SH

The *csn* gene was cloned into the pET-28(a^+^) vector with a C-terminal His tag and transferred into *E. coli* BL21 (DE3). Then, the recombinant protein was induced by different IPTG concentrations at temperatures ranging from 20°C to 35°C. The results indicated that Csn-SH showed its maximum enzymatic activity in the presence of 0.1 mmol/L IPTG at 20°C and 0.05 mmol/L IPTG at 30°C, respectively (Figure 1C). Because the IPTG is a potentially toxic chemical for cell growth (Zheng et al., 2021), the condition of 0.05 mmol/L IPTG at 30°C was chosen for further study. After purification in a Ni-NTA column, the enzymatic activity of Csn-SH increased from 29.04 to 366.14 U/mg. Compared with other chitosanases of the GH46 family heterologously expressed in *E. coli* BL21 (DE3), the enzymatic activity of Csn-SH was close to that of CsnQ from *Bacillus* sp. Q1098 (371.6 U/mg) (Ma et al., 2020) and much higher than that of Csn-BAC from *Bacillus* sp. MD-5 (41.67 U/mg) (Yang et al., 2020) and lower than CsnS from *Serratia* sp. QD07 (426.7 U/mg) after 60-h fermentation (Zheng et al., 2021). This indicated that the enzymatic activity of Csn-SH might be much higher after high density fermentation.

SDS-PAGE results showed that the Mw of purified Csn-SH was approximately 27.0 kDa (Figure 1D), which was in accordance with the theoretical Mw (27.4 kDa). In previous studies, the Mw of chitosanases from GH46 and GH75 families has mostly been found to range from 23.0 to 35.0 kDa, whereas chitosanases from GH3 and GH8 families usually exhibited an Mw greater than 40.0 kDa (Table 1). Furthermore, the Mw of Csn-SH was similar to those of chitosanases from *Bacillus*.

**TABLE 1:** The characteristics of chitosanases from different sources.

Name	Sources	Family	Mw (kDa)	pH	Temperature (°C)	Major products	References
Chitosanase	*Anabaena fertilissima*	GH3	41.0	–	–	DP2–3	Gupta et al., 2010
Chitosanase	*Bacillus* sp. TS	GH8	47.0	5.0	60	DP3–6	Zhou et al., 2015
Csn-SH	*Bacillus atrophaeus BSS*	GH46	27.0	5.0	45	DP2–4	This study
Csn	*Bacillus subtilis* 168	GH46	30.0	5.5	50	DP2–4	Pechsrichuang et al., 2013
BaCsn46A	*Bacillus amyloliquefaciens*	GH46	29.7	6.0	50	DP2–10	Qin et al., 2018
Csn-CAP	*Staphylococcus capitis*	GH46	35.0	7.0	30	DP2–3	Sun et al., 2018
CsnB	*Bacillus* sp. BY01	GH46	30.0	5.0	35	DP2–3	Yang et al., 2019
Csn-BAC	*Bacillus* sp. MD-5	GH46	35.0	7.0	40	DP2–3	Yang et al., 2020
CsnQ	*Bacillus* sp. Q1098	GH46	30.0	5.3	60	DP2	Ma et al., 2020
BaCsn46B	*Bacillus amyloliquefaciens* ECU08	GH46	29.0	6.5	55	DP2–3	Luo et al., 2020
CSN4	Marine mud metagenomic DNA	GH46	26.0	7.0	30	DP2–4	Sun et al., 2020
BbCSN-1	*Beauveria bassiana*	GH75	33.0	5.0	30	DP2–3	Liu et al., 2020
Csn75	*Aspergillus fumigatus* CJ22-326	GH75	23.5	5.0	55	DP2–4	Zhou et al., 2020

### Characteristics of Csn-SH

**FIGURE 2:**
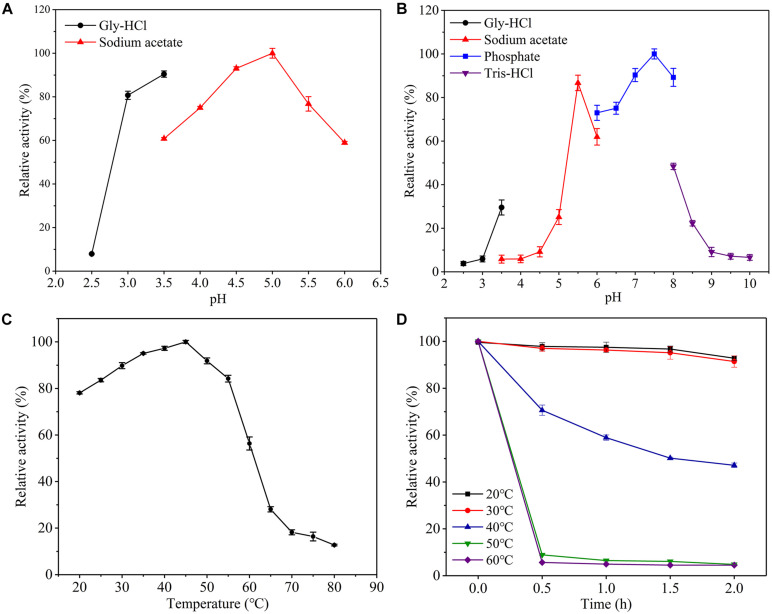
Effect of pH and temperature on the activity and stability of the purified Csn-SH. **(A,B)** Optimal pH and pH stability. **(C,D)** Optimal temperature and thermostability. The initial catalytic activity without preincubation was used as control, which was defined as 100%.

Chitosan precipitates in solution when the pH was greater than 6.0, so buffer with a pH lower than 6.0 was selected for the optimal pH study. The optimal pH levels for chitosanases were reported in previous studies mainly between 4.0 and 8.0 (Table 1), and the optimal pH of Csn-SH was 5.0 (Figure 2A). The activity of Csn-SH remained greater than 86.70% of its maximum value at pH 5.5 and 89.35% at pH 7.0 to 8.0 under 1 h of incubation at 45°C (Figure 2B). Additionally, Csn-SH showed high activity at temperatures ranging from 20 to 55°C and exhibited the maximum activity at 45°C. Notably, 78.11 and 83.57% of the maximum activity were observed at 20 and 25°C, respectively (Figure 2C). After incubation for 0.5 to 2 h, Csn-SH retained more than 92.84 and 91.43% of its original activity at 20 and 30°C, respectively. However, it was rapidly inactivated at 40°C, retaining 58.92% of its activity when incubated at 40°C for 1 h (Figure 2D). In particular, Csn-SH was stable after incubation at 30°C for 2 h, whereas CsnB from *Bacillus* sp. BY01 lost 50% of its activity when incubated at 30°C for 20 min (Yang et al., 2019), and CsnS from *Serratia* sp. QD07 retained 80.0% of its initial activity at 30°C for 2 h (Zheng et al., 2021).

In order to maximize enzyme activity and utilization efficiency, chitosanase is stable in high temperature, and alkaline conditions are often selected in industry. However, few chitosanases could meet the requirements. Wang et al. (2018) immobilized chitosanase on the surface of Fe_3_O_4_–SiO_2_ magnetite nanoparticles through covalent binding, which not only significantly increased the catalytic efficiency, but also improved the thermostability 11.0% and recycling times of chitosanase (Wang et al., 2018). Similar results were reported for amylase (Atiroglu et al., 2021). In addition, site-directed mutation (Yun et al., 2006) and fusion with related protein modules (Han et al., 2017) have been shown to improve the thermal stability of chitosanase as well. In this study, Csn-SH showed an optimal temperature at 45°C, and it was stable at pH 7.0 to 8.0. Thus, Csn-SH is a promising candidate for COSs preparation in industrial scale by improving its thermostability in further study.

The effects of different metal ions and chemical (1 and 5 mM final concentrations) on the activity of Csn-SH were also determined (Table 2); 1 and 5 mM Mn^2 +^ dramatically enhanced the activity of Csn-SH by 47.1 to 64.6%, respectively; 5 mM Mn^2 +^ enhanced the activity of CsnB by 2.6-fold with respect to its initial activity (Yang et al., 2019), and 10 mM Mn^2 +^ increased the activity of Csn21c 2-fold (Guo et al., 2019). The activities of chitosanases could be enhanced by Mn^2 +^ at different concentrations. The reason might be related to the different strain sources of chitosanases and the metal ion–binding sites in the chitosanase structure (Nguyen et al., 2014; Yang et al., 2019). However, most of the metal ions inhibited the activity of Csn-SH. In this study, the inhibitory effects of Cu^2 +^, Zn^2 +^, Fe^3 +^, and Al^3 +^ on Csn-SH activity were significantly greater than those of Na ^+^, Li ^+^, Mg^2 +^, Fe^2 +^, Co^2 +^, and Pb^2 +^. Cu^2 +^ caused the strongest inhibition of Csn-SH enzymatic activity (Table 2). The differences in the inhibitory effects of different metal ions on Csn-SH activity might be associated with the presence of a larger number of amino groups on the chitosan surface, which complexed with transition metal ions and thus decreased the enzyme activity (Brunel et al., 2013). In addition, 1 and 5 mM EDTA inhibited the activity of Csn-SH by 15.10 and 25.04%, respectively (Table 2), possibly because the –COOH group of EDTA and –NH_2_ on the chitosan surface can form an amide bond in a concentration-dependent manner in acidic solution (Gyliene et al., 2006). In accordance with our results, 5 mM EDTA has been shown to dramatically inhibit the activities of CsnB (*Bacillus* sp. BY01) (Yang et al., 2019) and CsnQ (*Bacillus* sp. Q1098) (Ma et al., 2020).

**TABLE 2:** Effects of different chemicals on the enzyme activity of purified Csn-SH.

Chemicals	Relative activity (%)	
	1 mM	5 mM
Control	100 ± 3.07	100 ± 1.04
Na^+^	80.38 ± 0.50	86.51 ± 1.80
K^+^	90.41 ± 1.50	101.25 ± 3.53
Li^+^	79.57 ± 2.44	88.17 ± 3.53
Mg^2+^	93.73 ± 1.82	95.85 ± 3.81
Ca^2+^	96.12 ± 2.32	97.65 ± 2.01
Cu^2+^	0 ± 0	0 ± 0
Mn^2+^	147.14 ± 3.70	164.55 ± 3.46
Zn^2+^	41.08 ± 1.82	11.37 ± 0.42
Fe^2+^	82.76 ± 1.00	72.26 ± 0.55
Co^2+^	81.57 ± 0.56	92.05 ± 0.28
Pb^2+^	85.90 ± 0.01	88.24 ± 4.36
Fe^3+^	23.53 ± 1.44	7.08 ± 2.84
Al^3+^	57.32 ± 3.38	9.71 ± 2.84
EDTA	84.90 ± 2.13	74.96 ± 4.15

### Substrate Specificity and Kinetic Parameters of Csn-SH

Colloidal chitin, CMC, colloidal chitosan (DD ≥ 85%), and water-soluble chitosan (DD ≥ 95%) were used as substrates to investigate the substrate specificity of Csn-SH. The results demonstrated that the hydrolytic activity of Csn-SH toward chitosan was directly proportional to the DD (Supplementary Table 1). Nevertheless, Csn-SH exhibited no activity toward chitin or CMC (Supplementary Table 1), which was in accordance with the reported findings for other chitosanases, such as Csn-BAC from *Bacillus* sp. MD-5 (Yang et al., 2020) and Csn-CAP from *Staphylococcus capitis* (Sun et al., 2018).

The kinetic parameters of Csn-SH were determined under the optimal conditions. The results presented in Supplementary Figure 1 showed that the *K*_*m*_ and *V*_*max*_ values of Csn-SH were 0.50 mg/mL and 140.05 μmol mg^–1^ min^–1^, respectively. The *K*_*m*_ and *V*_*max*_ of Csn21c from *Streptomyces albolongus* were 7.4 mg/mL and 263.1 μmol mg^–1^ min^–1^, respectively (Guo et al., 2019), and the corresponding values of Csn from *B. subtilis* were 1.57 mg/mL and 8.83 μmol mg^–1^ min^–1^, respectively (Pechsrichuang et al., 2013). The *K*_*m*_ value of Csn-SH was much lower than those of the above chitosanases, and the *V*_*max*_ was higher than those of these chitosanases, which indicated that Csn-SH presented high substrate affinity and catalytic efficiency (Zhou et al., 2020).

### Hydrolysis Pattern of Csn-SH

**FIGURE 3:**
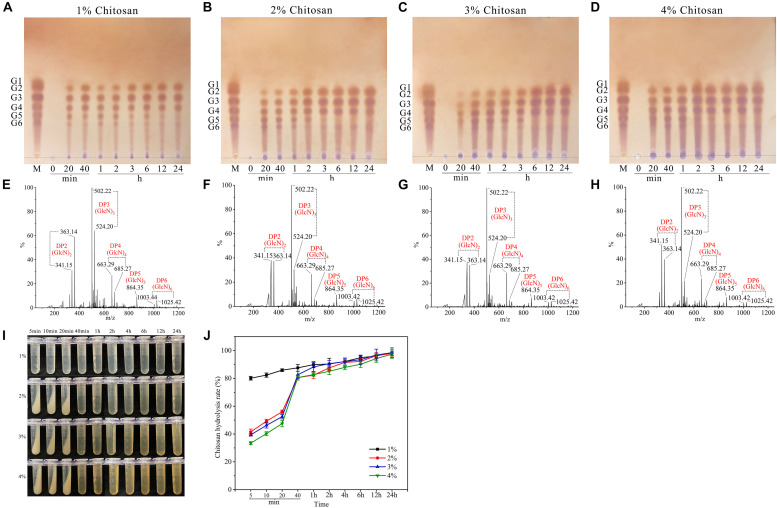
TLC, ESI-MS analysis, and yield of chitosan hydrolysates prepared with Csn-SH. **(A–D)** TLC and ESI-MS analysis of 1–4% (wt/vol) chitosan hydrolysates for different reaction time, respectively. G1–G6 indicated (GlcN)_1_ to (GlcN)_6_. Lane M: standard chitooligomers. Lanes 0–24: Chitosan hydrolysates prepared with Csn-SH incubated at 45°C for 20 min, 40 min, 1, 2, 3, 6, 12, and 24 h, respectively. **(E–H)** ESI-MS analysis of 1–4% chitosan hydrolysates prepared with Csn-SH at 45°C for 24 h, respectively. **(I)** The precipitation of undegraded chitosan with 20 U/mL crude Csn-SH at different reaction time. **(J)** The hydrolysis rates of 1–4% chitosan by Csn-SH at different reaction time with 20 U/mL crude Csn-SH.

The hydrolysis properties of Csn-SH toward colloidal chitosan (1–4%, wt/vol) were analyzed by TLC and ESI-MS (Figure 3). In 20 min, Csn-SH could effectively hydrolyze 1–4% chitosan to yield COSs [mainly of (GlcN)_2__–__6_]. With extension of the reaction time, DP5 and DP6 were further degraded into DP2–4, whereas DP4 was not hydrolyzed further (Figures 3A–D). The hydrolysates of chitosan after 24 h of incubation at 45°C were also detected. ESI-MS analysis showed that 1–4% of the chitosan hydrolysates mainly consisted of (GlcN)_2__–__4_, with a small amount of (GlcN)_5–6_ (Figures 3E–H), which was consistent with the TLC results.

To shed light on the cleavage pattern of Csn-SH, (GlcN)_2__–__6_ were degraded by Csn-SH, and the hydrolysates were also analyzed by TLC. The results showed that Csn-SH could not hydrolyze (GlcN)_2__–__4_ (Supplementary Figures 2A–C), whereas (GlcN)_5_ could be degraded into (GlcN)_2__–__3_ (Supplementary Figure 2D). For (GlcN)_6_ substrates, (GlcN)_2__–__4_ products were obtained (Supplementary Figure 2E). CSN4 from a marine mud metagenome could degrade (GlcN)_6_ into (GlcN)_2_ and (GlcN)_4_ without (GlcN)_3_, which demonstrated that CSN4 preferred to digest substrates asymmetrically rather than symmetrically (Sun et al., 2020). Therefore, the above results indicated that Csn-SH exhibits no preference for substrate symmetry. Additionally, the hydrolysis properties of Csn-SH were similar to those of other members of GH46 family chitosanases, such as Csn from *B. subtilis* 168 (Pechsrichuang et al., 2013) and Csn-CAP from *S. capitis* (Sun et al., 2018), which yielded COSs with DP2–3 or DP2–4 (Table 1). These results revealed that Csn-SH could hydrolyze chitosan in an endo-type pattern and recognize (GlcN)_5_ as the minimal substrate.

### Economic and Efficiency Analysis of Csn-SH

It is worth noting that the hydrolysis rate of 1–4% chitosan by Csn-SH (20 U/mL, final concentration) reached 80.57% within 40 min (Figure 3J). To evaluate whether Csn-SH is cleaner and more efficient than chitosanases and commercial enzymes reported previously, we compared the characteristics of chitosanases, commercial cellulase, lipase, papain, pectinase, and pepsin during the hydrolytic processes of chitosan; the examined characteristics included the dosage of enzymes, water consumption, and the value of hydrolysates and resulting profits (Table 3). The characteristics of the above enzymes were summarized according to previous publications (Lin et al., 2002; Cabrera and Van Cutsem, 2005; Roncal et al., 2007; Lee et al., 2008; Tegl et al., 2016; Zhou et al., 2020). One ton of colloidal chitosan was used as the raw material, and the corresponding concentration was obtained by using 1% acetic acid. The profits were calculated based on the raw chitosan materials and the yield of COSs, except for the costs of water and enzymes. The conversion of chitosan and COS prices was calculated based on the data (Deng et al., 2020). The results presented in Table 3 showed that Csn-SH produced the highest profits during the degradation of chitosan, followed by lipase, pepsin, Csn75, and cellulase. The output values of the COSs prepared with pectinase and papain were lower than the input values of the chitosan material. It was noteworthy that among these enzymes, Csn-SH took only 40 min to complete the same process, for which lipase and pepsin required 6 and 20 h, respectively. In addition, the amount of water consumption by Csn-SH was also the lowest, at only 25% and 50% of the amounts required by lipase and pepsin, respectively (Table 3). The above results indicated that Csn-SH could reduce the waste of water resources and decrease hydrolysis time during the degradation of chitosan relative to other enzymes.

**TABLE 3:** The properties and profits of different enzymes for the conversion of chitosan to chitooligosaccharides (COSs).

Enzyme	Characteristics					Inputs/(1-ton chitosan)	Outputs/COSs^1^	Profits (CNY^2^)	Sources
	Dosage	Chitosan	Time	Hydrolysis rate	DP of products				
Csn-SH	1% (20 U/mL)	4%, 85% DD	40 min	80.57%	2–6	250 kg Csn-SH, 25 tons water	806 kg	268,494	This study
Csn75	30 U/mL	2%, 95% DD	2 h	40.00%	2–6	1.5 × 10^9^ U Csn75, 50 tons water	400 kg	45,600	Zhou et al., 2020
Commercial cellulase	0.25%	0.5%, 87% DD	24 h	40.00%	2–6	500 kg cellulase, 200 tons water	400 kg	45,600	Tegl et al., 2016
Commercial lipase	0.5%	0.5%, 83% DD	6 h	58.20%	1–6, ≥6	1 ton lipase, 200 tons water	582 kg	145,518	Lee et al., 2008
Commercial papain	0.3%	1%, 87% DD	24 h	11.07%	3–7	300 kg papain, 100 tons water	111 kg	–	Lin et al., 2002
Commercial pectinase	10%	1%, 88% DD	24 h	17.00%	6–11	10 tons pectinase, 100 tons water	170 kg	–	Cabrera and Van Cutsem, 2005
Commercial pepsin	1%	1%, 93% DD	20 h	52.20%	Average DP 16.6	1 ton pepsin, 100 tons water	522 kg	112,578	Roncal et al., 2007

*^1^The price of chitosan and COSs were 174 and 550 CNY/kg, respectively. ^2^CNY indicates China Yuan. The profit was the COSs’ values minus the chitosan costs.*

### Homology Modeling and Molecular Docking

**FIGURE 4:**
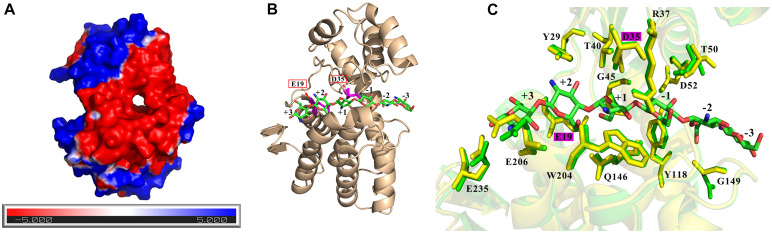
Homology modeling structure and docking of Csn-SH. **(A)** The electrostatic-potential surface of Csn-SH. **(B)** The overall structural view of Csn-SH in complex with (GlcN)_6_. (GlcN)_6_ was labeled −4 to + 2 from the nonreducing end to the reducing end. Two catalytic residues (E19 and D35) of Csn-SH were colored by magenta. **(C)** Substrate interaction of Csn-SH. The residue numbers of Csn-SH represented the sequence without 32 amino acids (signal peptide) at N-terminal. The amino residues from Csn-SH and chitosanase CsnMY002 (PDB: 7C6C) are colored by yellow and green, respectively.

A three-dimensional model of Csn-SH was generated by homology modeling using the crystal structure of *B. subtilis* MY002 (PDB: 7C6C; identity: 91.3%) as a template (Li et al., 2021). Chitosan is a natural cationic polysaccharide, so the substrate-binding sites of chitosanase are highly negatively charged (Takasuka et al., 2014). As shown in Figure 4A, the substrate-binding region of Csn-SH formed a highly negatively charged closed tunnel (Figure 4A), which differed to the open clefts structure of other GH46 chitosanases (Marcotte et al., 1996; Li et al., 2021). Tunnel-like substrate-binding sites lined with aromatic residues often exhibited in the structure of processive enzymes, which could provide a hydrophobic sheath to facilitate substrate sliding in the tunnel (Varrot et al., 2003; Li et al., 2021). Conserve domain database analysis revealed that Csn-SH was a monomeric enzyme belonging to the lysozyme superfamily. It was composed of two lobes connected by a long bent α-helix and a hinge segment (Figure 4B). The results were similar to those obtained for CsnMY002 from *B. subtilis* (Li et al., 2021), SACTE_5457 from *Streptomyces* (Takasuka et al., 2014), and BaCsn46B from *B. amyloliquefaciens* (Luo et al., 2020).

Molecular docking analysis was performed to further explore the interaction of Csn-SH and (GlcN)_6_. The substrate was located in the closed tunnel of Csn-SH, and (GlcN)_6_ was labeled −4 to +2 from the nonreducing end to the reducing end (Figure 4B). Two highly conserved catalytic residues, E19 and D35, were identified as the general acid/base and nucleophilic catalytic residues of Csn-SH, respectively, which were located on either side of the substrate (Figure 4B). During the reaction, E19 protonated glycosidic oxygen, and D35 polarized the attacking water to complete hydrolysis by using an “inverting” catalytic mechanism (Marcotte et al., 1996). The presumptive cleavage site of Csn-SH was matched to the glycosidic bond between the −1 to +1 subsite (Figure 4B), which suggested that Csn-SH might cleave (GlcN)_6_ into (GlcN)_2_ and (GlcN)_4_. This speculation was confirmed by the result of TLC, which showed that the hydrolysates prepared with Csn-SH contained (GlcN)_2_ and (GlcN)_4_. It was noteworthy that (GlcN)_3_ was also found in the hydrolysates and considered as the dominant product (Figure 3 and Supplementary Figure 2E). The results implied that another putative cleavage site might locate between the −2 and −1 position (Figure 4C); thus, Csn-SH could cleave (GlcN)_6_ into two (GlcN)_3_ molecules. Furthermore, the docking and TLC results of Csn-SH were consistent with the results of chitosanase OU01 (Lyu et al., 2014) and CsnMY002 (Li et al., 2021). However, the hydrolysates from CsnMY002 mainly contained (GlcN)_2_ and (GlcN)_3_ without (GlcN)_4_, which might be related to the difference of enzyme dosage in the reaction mixture.

In addition, results from Figure 4C showed that the amino acid residues and catalytic residues in Csn-SH superimposed well with the corresponding residues in the CsnMY002 (PDB: 7C6C) structure. The sugar units and adjacent residues (Y29, R37, G45, T50, D52, Y118, Q146, G149, W204, E206, and E235) could form a hydrogen-bond network to stabilize the substrate (Li et al., 2021). Moreover, the hydrogen bonding between Y29, Y118, W204, and the sugar units might inhibit the substrate sliding in the substrate-binding tunnel (Zakariassen et al., 2009). Nevertheless, the functions of these corresponding key residues in Csn-SH remained to be further investigated. The above results proposed that Csn-SH might cleave glycosidic bonds of chitosan between subsites −2 and +1 as a nonprocessive enzyme (Lyu et al., 2015; Li et al., 2021). However, more studies need to explore the action mode.

### Antifungal Activity of COSs

**FIGURE 5:**
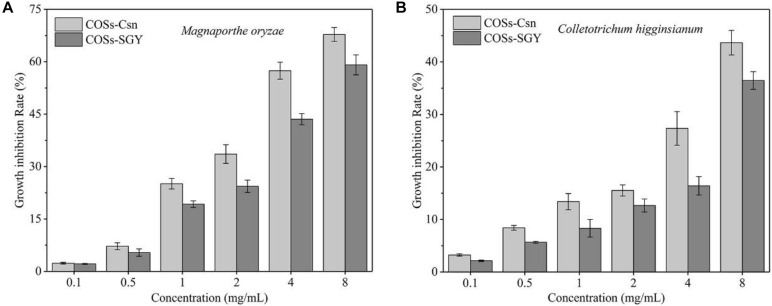
The fungal mycelia generation inhibition rate affected by COSs at different concentrations. **(A)** Inhibition of mycelia generation in *M. oryzae* treated with COSs at different concentrations. **(B)** Inhibition of mycelia generation in *C. higginsianum* treated with COSs at different concentrations. COSs-Csn indicated the COSs prepared with Csn-SH, whereas COSs-SGY represented the COSs purchased from Guangzhou SGY Agricultural Science and Technology Co., Ltd.

The inhibition of COSs on phytopathogenic fungi *M. oryzae* and *C. higginsianum* are shown in Figure 5. *M. oryzae* and *C. higginsianum* have been reported to cause severe damage to rice (Griffith et al., 2021) and various cruciferous plants, respectively (Yan et al., 2020). COS treatments significantly inhibited the growth of the *M. oryzae* and *C. higginsianum*, but had a stronger inhibitory effect on *M. oryzae*. The inhibition effects became more significant as the concentration increases, which displayed a dose-dependent inhibition (Figure 5). The EC_50_ of *M. oryzae* and *C. higginsianum* with COSs-Csn treatment were 4.84 and 8.47 mg/mL, respectively, while the values with COSs-SGY treatment were 6.06 and 9.91 mg/mL, respectively. These results were consistent with previous study showing that COSs exhibited good antifungal activity (Wang et al., 2021b), because the cationic -NH_2_ groups of COSs can form ammonium groups by absorbing H ^+^, and then bind to negatively charged components of the microbial cell wall, resulting in agminated microbes and further lysis (Liaqat and Eltem, 2018).

However, antifungal activity of COSs is significantly affected by the DP, degree of acetylated and microorganism types (Liaqat and Eltem, 2018). Among them, DP ≥ 5 was essential for antimicrobial activity of fully deacetylated COS (Li et al., 2014). The growth inhibition rate (27.35%) of *M. oryzae* with 4 mg/mL COSs-Csn treatment (Figure 5A) was higher than CoA-COSs (20.0%) in a previous study (Wang et al., 2021b); the reason may be related to the difference of DP. COSs-Csn was composed of DP2–6, whereas CoA-COSs consisted of DP3–5. However, the antifungal activity of COSs-SGY (DP2–7) was lower than COSs-Csn (DP2–6), which might be due to the relative content of oligosaccharides with different DP (Sanchez et al., 2017). Previous research pointed out that COSs (DP2–6) could restore the activities of endogenous antioxidants and inhibit intracellular ROS, which played great potential for the oxidative disease treatment (Liu et al., 2009). Therefore, COSs prepared with Csn-SH have broad application prospects in the fields of antifungal and antioxidative agents, and other active functions need to be further studied.

## Conclusion

Our results suggested that Csn-SH is a biotechnologically potential chitosanase characterized from *B. atrophaeus* BSS. Csn-SH showed optimal activity under the condition of 45°C and pH 5.0, and it was stable at pH 7.0–8.0, below 30°C. (GlcN)_5_ was the minimally recognized substrate of Csn-SH. Under the optimal reaction condition, Csn-SH hydrolyzed colloidal chitosan (concentration ≥1%) into DP2–6 in an endo-type pattern. Importantly, Csn-SH could reduce the waste of water resources and save hydrolysis time during the hydrolytic processes of chitosan. The hydrolysates prepared with Csn-SH could inhibit the growth of fungi. The excellent hydrolytic capabilities make Csn-SH a perfect candidate for the efficient production of COSs that could be used as biocontrol agents.

## Data Availability Statement

The datasets presented in this study can be found in online repositories. The names of the repository/repositories and accession number(s) can be found below: https://www.ncbi.nlm.nih.gov/genbank/, CP007640.

## Author Contributions

DC: investigation, data curation, analysis, writing-original draft preparation, and conceptualization. JY: investigation, review and editing, and conceptualization. BL: investigation. HS: conceptualization, project administration, review and editing, and funding acquisition. All authors have read and agreed to the published version of the manuscript.

## Conflict of Interest

The authors declare that the research was conducted in the absence of any commercial or financial relationships that could be construed as a potential conflict of interest.

## Footnotes

1 https://www.ncbi.nlm.nih.gov/orffinder/

2 http://www.cbs.dtu.dk/services/SignalP/

3 https://web.expasy.org/protparam/

4 http://www.ebi.ac.uk/Tools/msa/clustalo/

5 http://espript.ibcp.fr/ESPript/cgi-bin/ESPript.cgi

## Supplementary Material

Supplementary Figure 1 The Lineweaver–Burk plot of the Csn-SH activity.

Supplementary Figure 2 TLC analysis of (GlcN)_2__–__6_ hydrolysates prepared with Csn-SH.

Supplementary Table 1 Substrate specificity of purified Csn-SH.
