# Supplementary material for: Efficient Preparation of Chitooligosaccharide With a Potential Chitosanase Csn-SH and Its Application for Fungi Disease Protection
Source: Front Microbiol. 2021 Jun 17;12:682829. doi: 10.3389/fmicb.2021.682829 (PMC8249199; doi:10.3389/fmicb.2021.682829)
Supplement: Supplementary Figure 1 — The Lineweaver–Burk plot of the Csn-SH activity. [file Data_Sheet_1.DOCX]

Supplementary Material





**Supplementary Figure 1.** The Lineweaver-Burk plot of the Csn-SH activity. [S] indicated the concentration of chitosan. The linear fitting equation was y=0.239*ⅹ*+0.119, the $\frac{1}{Vm}$ equal to the intercept on axis y, and the $-\frac{1}{Km}$ equal to the intercept on axis *x*.


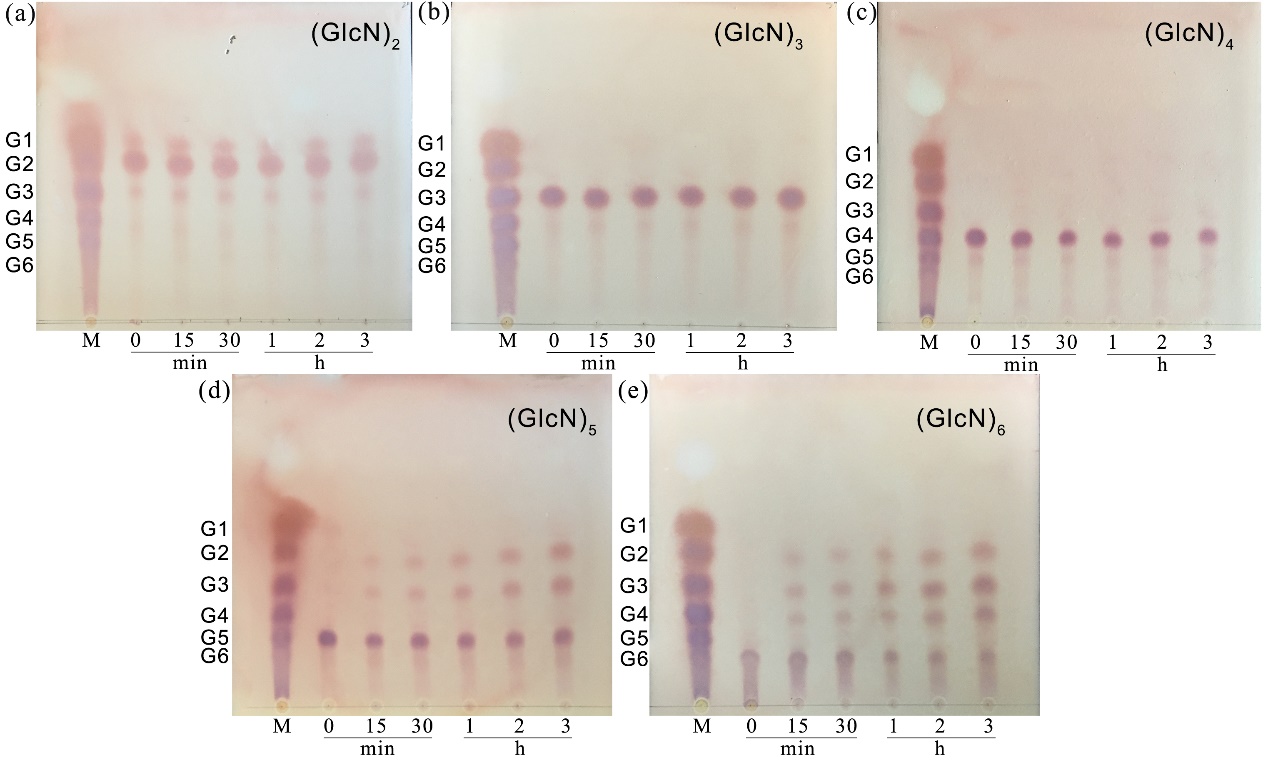


**Supplementary Figure 2.** TLC analysis of hydrolysates of (GlcN)_2-6_ prepared by Csn-SH. G1-G6 indicated (GlcN) to (GlcN)_6_. Lane M: standard chitooligomers. Lanes 0-3: Hydrolysates of standard chitooligomer incubated at 45 ℃ for 15 min, 30 min, 1 h, 2 h and 3 h, respectively.

**Supplementary Table 1**. Substrate specificity of purified Csn-SH.

| Substrate | DD (%) | Relative activity (%) |
| --- | --- | --- |
| Chitin | 0 | 0 |
| Cellulose | - | 12.3+0.7 |
| Chitosan | 85% | 100.0+1.6 |
| Chitosan | 95% | 108.0+2.4 |

DD: degree of deacetylation
